# Role of 5-HT1A-mediated upregulation of brain indoleamine 2,3 dioxygenase 1 in the reduced antidepressant and antihyperalgesic effects of fluoxetine during maintenance treatment

**DOI:** 10.3389/fphar.2022.1084108

**Published:** 2022-12-16

**Authors:** Jiajia Dai, Hyangin Kim, Zerong You, Michael F. McCabe, Shuzhuo Zhang, Shiyu Wang, Grewo Lim, Lucy Chen, Jianren Mao

**Affiliations:** ^1^ MGH Center for Translational Pain Research, Department of Anesthesia, Critical Care and Pain Medicine, Massachusetts General Hospital, Harvard Medical School, Boston, MA, United States; ^2^ Department of Anesthesiology, Xiangya Hospital, Central South University, Changsha, Hunan, China

**Keywords:** fluoxetine, 5-HT 1A auto-receptor, indoleamine 2,3 dioxygenase 1, depression, chronic pain

## Abstract

The reduced antidepressant and antihyperalgesic effects of selective serotonin reuptake inhibitors (SSRIs) such as fluoxetine during maintenance treatment has been reported, but little is known about the molecular mechanism of this phenomenon. In three comorbid pain and depression animal models (genetic predisposition, chronic social stress, arthritis), we showed that the fluoxetine’s antidepressant and antihyperalgesic effects were reduced during the maintenance treatment. Fluoxetine exposure induced upregulation of the 5-hydroxytryptamine 1A (5-HT1A) auto-receptor and indoleamine 2,3 dioxygenase 1 (IDO1, a rate-limiting enzyme of tryptophan metabolism) in the brainstem dorsal raphe nucleus (DRN), which shifted the tryptophan metabolism away from the 5-HT biosynthesis. Mechanistically, IDO1 upregulation was downstream to fluoxetine-induced 5-HT1A receptor expression because 1) antagonism of the 5-HT1A receptor with WAY100635 or 5-HT1A receptor knockout blocked the IDO1 upregulation, and 2) inhibition of IDO1 activity did not block the 5-HT1A receptor upregulation following fluoxetine exposure. Importantly, inhibition of either the 5-HT1A receptor or IDO1 activity sustained the fluoxetine’s antidepressant and antihyperalgesic effects, indicating that 5-HT1A-mediated IDO1 upregulation in the brainstem DRN contributed to the reduced antidepressant and antihyperalgesic effects of fluoxetine. These results suggest a new strategy to improving the therapeutic efficacy of SSRI during maintenance treatment.

## Introduction

Depression is a comorbid condition of chronic pain (Tunks et al., 2008; Lee et al., 2018; Serafini et al., 2020). Chronic pain is often managed by medications such as opioid analgesics and antidepressants for symptomatic relief (Cohen et al., 2021). Opioids are strong analgesics but with serious side effects including abuse and addiction. Antidepressants including tricyclics and selective serotonin and/or norepinephrine reuptake inhibitors (SSRI or SNRI) are effective in chronic pain treatment (Mao, 2017; Bonilla-Jaime et al., 2022), but diminishing response to successive doses of a drug occurs during maintenance treatment (Fornaro et al., 2019; Fava, 2020). This phenomenon is more prominent with SSRI (e.g., fluoxetine) than SNRI and tricyclic antidepressants (Katz, 2011). However, little has been known regarding the molecular mechanism of the reduced antidepressant and antihyperalgesic effects of SSRI.

The 5-hydroxytryptamine 1A (5-HT1A) receptors are categorized as pre-synaptic (auto-receptors) and post-synaptic receptors that differentially regulate the effect of antidepressants (Albert et al., 2014). The 5-HT1A auto-receptor resides on the soma and dendrites of serotonergic neurons in the brainstem raphe nuclei. Activation of 5-HT1A auto-receptors reduces the firing rate of serotonergic neurons thereby regulating synaptic 5-HT levels in their projection areas. 5-HT1A auto-receptors are known to play a role in mood disorders and their treatments (Salaciak and Pytka, 2021; Schneck et al., 2021). Conditional knockdown of 5-HT1A auto-receptors in the brainstem dorsal raphe nucleus (DRN) increased the antidepressant response as well as the 5-HT release into the prefrontal cortex (Ferres-Coy et al., 2013). An increase in 5-HT1A auto-receptor density in the DRN attenuated the therapeutic activity of SSRIs and, conversely, desensitization of these receptors promoted serotonergic transmission in the brain (Rainer et al., 2012).

Biosynthesis of 5-HT in the brain is regulated by two rate-limiting enzymes of tryptophan metabolism: indoleamine 2,3 dioxygenase 1 (IDO1) and tryptophan hydroxylase 2 (TPH2) (Hoglund et al., 2019). TPH2 is responsible for 5-HT production, whereas IDO1 is located in the opposite side of the tryptophan metabolic pathway away from 5-HT production. Recent studies have shown that the IDO1 isoform is expressed in the brain (Kwidzinski et al., 2003), which can be induced by inflammatory mediators such as TNFα, IFN-γ and interleukins (Duda et al., 2019; Sundaram et al., 2022). Our previous study demonstrated that upregulation of IDO1 shifted tryptophan metabolism away from 5-HT biosynthesis, contributing to the development of depression in chronic pain condition (Kim et al., 2012). However, the role of IDO1 in the reduced antidepressant and antihyperalgesic effects of SSRI is unclear.

In the present study, we used three depression-pain comorbidity animal models (genetic predisposition, chronic social stress, arthritis) to examine the hypothesis that the 5-HT1A auto-receptor is critical for the reduced antidepressant and antihyperalgesic effects of fluoxetine through upregulation of IDO1 expression in the DRN.

## Materials and methods

### Experimental animals

Male WKY and Wistar rats (250–300 gm; Charles River), 7 to 10-week-old male B6N(Cg)-*Htr1a* ^*tm1.1(KOMP)Vlcg*^/J (5-HT1A^−/−^) and C57BL/6NJ wild (WT) mice (Jackson Laboratory) were used. Animals were housed individually (21°C ± 2°C, relative humidity 50 ± 10%, 12 h light/dark cycle) with free access to water and food. Research protocols were approved by the Massachusetts General Hospital Institutional Animal Care and Use Committee. Experiments were conducted with the experimenter being unaware of group assignment.

### Drugs

Fluoxetine hydrochloride, the 5-HT1A receptor antagonist (*N*-[2-[4-(2-methoxyphenyl)-1-piperazinyl [ethyl]- *N*-(2-pyridyl) cyclohexane carboxamide (WAY100635)), and the IkB kinase inhibitor (2-amino-6-[2-(cyclopropylmethoxy)-6-hydroxylphenyl]-4-piperidin-4-yl-nicotinonitrile (ACHP)) were purchased from Tocris Bioscience. The IDO1 inhibitor L-1-methyl-tryptophan (1-MT) was from Aldrich-sigma. Fluoxetine and WAY100635 were dissolved in sterilized PBS, and PBS was used as vehicle control. ACHP was dissolved in 10% dimethyl sulfoxide (DMSO, Sigma-Aldrich) to prepare stock solutions (1 mM) and then diluted in PBS to 1μM, thus the final 0.01% DMSO in PBS was used as vehicle control. 1-MT was initially dissolved in 0.5 N HCl at 50 mg/ml, adjusted to 10 mg/ml with PBS, and PBS was used as control.

### Surgical procedures

#### Hindpaw monoarthritis

Hindpaw monoarthritis was induced by a single injection (50 μL/rat, 20 μL/mouse) of 1 mg/ml complete Freund’s adjuvant (CFA) (Sigma Aldrich, St. Louis, MO) into a unilateral tibio-tarsal joint cavity under brief isoflurane anesthesia (Kim et al., 2012). PBS was administered to the control group. Local redness and joint swelling were present in CFA groups but not in control group during the experimental period.

#### Brain cannula implantation and drug administration

Under brief isoflurane anesthesia, a guide cannula (26 gauge, Plasticsone) was implanted just above the DRN (on midline, 7.3 mm posterior and to 6.0 mm ventral to bragma). An injection needle (33 gauge, Plasticsone) was inserted through the guide cannula. A drug solution or vehicle (1 μL) was slowly injected over 5 min using a Hamilton syringe.

### Chronic social stress model

To induce depression-like behavior in rats, chronic social stress was introduced using a modified resident-intruder social interaction method as described previously (Kim et al., 2012). An experimental rat (275–300 gm), named as an intruder rat in this model, was transferred from its home cage into a cage of a resident rat (500–600 gm) for 1 h per day. The intruder rat and the resident rat were separated by a small round wire-mesh compartment (diameter 11 cm, height 14 cm) within the resident cage. After 1 h, the intruder rat was returned to its home cage. This procedure was carried out at the beginning of the dark cycle in a 24-h light cycle (light on and off for each 12 hour-period). An intruder rat was confronted with a different resident rat each day. This process lasted for 4 weeks. For controls, rats were placed in the same behavioral room but without the social interaction with a resident rat.

### Behavioral tests

#### Thermal hyperalgesia test (TH)

Paw withdrawal latency (PWL) was tested using a plantar test instrument (Ugo Basile, Model 07370, ITA). The key testing procedures are as follows. First, prior to the test, the animals were acclimated individually to a plastic chamber which was placed on the surface of a glass plate (2 mm thickness) for half an hour. Next, three heat stimuli, generated by a radiant heat stimulator, were applied to the plantar area of each hind-paw repeatedly at 5-min intervals. The PWL was automatically recorded when the hind paw moved. To avoid tissue damage, a 30 s cutoff was pre-set.

#### Forced swimming test (FST)

Forced swimming test is a most commonly used assay performed for studying depression-like behavior in rodents. As described previously (Dai et al., 2019), animals were placed in a transparent cylindrical glass container filled with tap water (22°C–24°C). After being forced to swim 6 min, the durations of time they spent as being immobile were recorded in the last 5 min.

#### Tail suspension test (TST)

According to the method of Steru et al. (1985), a mouse was suspended on a 50-cm-height rod, using adhesive tape placed 20 mm from the tip of its tail. The movements of the animal were recorded. The total duration of test (6 min) was divided into periods of agitation and immobility.

### RN 46A cell cultures

RN 46A cells (Public Health England), an immortalized serotonergic neuronal cell line derived from embryonic rat medullary raphe cells by infection with a retrovirus encoding the temperature-sensitive mutant of SV 40 large T-antigen (White et al., 1994), were cultured with 1:1 solution of DMEM/Ham’s F12 (GIBCO, Invitrogen), 100 U/ml penicillin, and 100 ug/ml streptomycin (Invitrogen) in a humidified atmosphere of 5% CO_2_ and 95% air at 33°C.

### Immunohistochemistry

Cells were fixed with 4% paraformaldehyde for 15 min at room temperature, rinsed with PBS. After fixation, cover cells with ice-cold 100% methanol, incubated cells for 10 min in freezer, rinsed with PBS and incubated in blocking solution (PBS containing 1% BSA and 0.3% Triton X-100) for 1 h. Cells were then incubated overnight at 4°C on a rocker with one of the following primary antibodies: 1:200 NF-κB p65 mouse monoclonal (Santa Cruz Biotechnology Inc.) (Cordoba-David et al., 2022; Zveik et al., 2022). Cells were then washed with PBS three times and incubated with 1:300 cyanine3-conjgated secondary antibody (Jackson Immuno Research Lab. Inc.) for 1 h at room temperature. Blue fluorescent DAPI (1:400; Invitrogen) was used to stain the nucleus of fixed cultured RN46A cells for 15 min. Moreover, for all antibodies used in the present study, we applied primary delete staining (only secondary antibody was used) to confirm the true positive. Images were examined with a fluorescence microscope (Olympus) and captured with a digital camera. The images were analyzed using Adobe PhotoShop (Version 7).

### Western blot

The DRN was collected and homogenized in an SDS lysis buffer (10 ml/mg tissue, ingredients of SDS buffer) containing a cocktail of protease and phosphatase inhibitors (Sigma, St. Louis, MO, United States of America) at 4°C. Samples (50 μg per lane) were separated by SDS-PAGE gel (4%–15% gradient gel; Bio-Rad) electrophoresis and then transferred to polyvinylidene difluoride filter (PVDF) membranes (Millipore, MA). Membranes were blocked with 5% nonfat dry milk in Tris buffered saline with Tween 20 (TBST) for 1 h at room temperature and incubated over night at 4°C on a rocker with one of following primary antibody: 1:1000 5-HT1A receptor (abcam; rabbit polyclonal antibody) (Chao et al., 2020; Kondaurova et al., 2020), 1:200 IDO1 (Santa Cruz Biotechnology Inc.; rabbit polyclonal antibody) (Kim et al., 2012), and 1:1000 NF-κB p65 (Santa Cruz Biotechnology Inc.; mouse monoclonal antibody). After being washed with TBST x 4, membranes were incubated with 1: 8,000 HRP conjugated secondary antibody (Millipore) at room temperature for 1 h. After washing, protein bands were visualized in enhanced Chemiluminescent (ECL) solution (Pierce) for 5 min and exposed to hyperfilms for 15 min. Blots were incubated in a Stripping buffer (Pierce) for 15 min at room temperature and reprobed with 1:12,000 anti-β-actin antibody (Sigma) as a loading control. Western blots were made in triplicates. Band density was measured and normalized against a loading control band.

### Real-time RT-PCR

Total RNA was isolated from each group of samples using TRIzol (Invitrogen, Grand Island, NY). Then, 4 μg of total RNA was used to prepare cDNA according to the protocol for SuperScript III kit (Invitrogen, Grand Island, NY). TaqMan Gene Expression Assays (Applied Biosystems) containing primers and probes for 5-HT1A (Rn00561409_s1) and IDO1 (Rn00675146_ m1) were used to quantify candidate genes. The reaction was performed under the cycling conditions required in the protocol for TaqMan with a 7300 Real-Time PCR System (Applied Biosystems). The expression of candidate genes was normalized to GAPDH to obtain a ΔCt value and to calculate a 2^−(mean ΔΔCt)^.

### HPLC

Contents of tryptophan, kynurenine, and serotonin in the DRN were determined by HPLC, as previously described (Kim et al., 2012). Samples were taken between 2 and 5 PM. Kynurenine was measured by a UV detector (Shimadzu, SPD-10Avp; 360 nm wavelength). Tryptophan and serotonin were measured by a fluorescence detector (Shimadzu, RF-10Axl; 286 nm excitation and 366 nm emission wavelength). External calibration was made daily to minimize day-to-day variations by using freshly prepared control samples of same concentration (100 nmol/L tryptophan, 100 μmol/L kynurenine, and 100 nmol/L 5-HT).

### Statistical analyses

**FIGURE 1:**
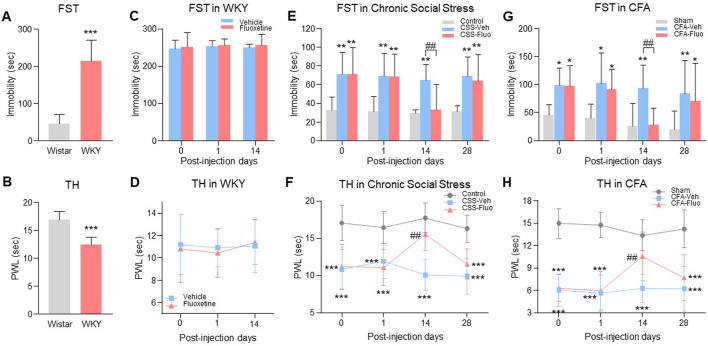
Rats with comorbid pain and depression displayed resistance to fluoxetine maintenance treatment. **(A)** WKY rats showed prolonged immobility time in FST as compared to the Wistar rats. **(B)** The decrease of thermal pain threshold in TH appeared in WKY rats. **(C)** Fluoxetine treatment did not change immobility time in FST on WKY rats, indicating the WKY rats were resistant to the fluoxetine effect. **(D)** Fluoxetine had no effect on PWL of WKY rats in TH compared with vehicle treatment group. **(E)** Chronic fluoxetine administration reversed the increase of immobility time in FST arising in chronic social stress rats on day 14 post injection, but failed on day 28. **(F)** Fluoxetine attenuated chronic social stress-induced PWL decreases on day 14, but not on day 28. **(G)** In the CFA model, fluoxetine also reversed the immobility time that was significantly increased by CFA in the FST on day 14, but not on day 28. **(H)** Like chronic social stress rats, CFA rats showed the same trends in TH. The data are presented as the mean ± SD (*n* = 8). *p < 0.05, **p < 0.01 and ***p < 0.001 compared to the Wistar rats, vehicle-treated WKY rats, Control in chronic social stress or Sham in CFA; ##*p* < 0.01, CSS-Veh versus CSS-Fluo and CFA-Veh versus CFA-Fluo. Sec indicates second; FST, forced swim test; TST, tail suspension test; TH, thermal test; PWL, paw withdrawal latency; CSS, chronic social stress; Veh, vehicle; Fluo, fluoxetine.

All data were expressed as mean ± SD. Sample size was estimated using power analysis based on the previous data on behavioral testing (Kim et al., 2012) and our pilot experiments. Behavioral tests at different time points in this study were performed within the same animals. For FST and TH in Figures 1A, B, unpaired 2-tailed Student t-test was used to compare differences between groups. For FST, TST and TH with different time points, repeated measures (RM) 2-way ANOVA and *post hoc* multiple comparison Bonferroni tests were used to compare the performances between groups in each time point. HPLC, western blot, real-time PCR and all other behavior data were analyzed using a 1-way or 2-way ANOVA and *post hoc* multiple comparison Bonferroni tests. The statistical analyses were performed using GraphPad (version 8.0) with the significance level set at 0.05.

## Results

### Reduced antidepressant and antihyperalgesic effects of fluoxetine during maintenance treatment

To examine whether the fluoxetine effects on comorbid pain and depression would be diminished during maintenance treatment, we used three depression-pain comorbidity models (genetic predisposition, chronic social stress, CFA-induced hindpaw monoarthritis). (1) Wistar-Kyoto (WKY) rats, a genetic model of depression and hyperalgesia (Pare, 1994), (Millard et al., 2020; Ferdousi et al., 2021), had depression-like behavior shown as increased baseline immobility time in forced swimming test (FST) (Figure 1A, unpaired t-test, *n* = 8, t = 7.743, df = 14, *p* < 0.0001) and hyperalgesia-like behavior shown as decreased baseline paw withdrawal latency (PWL) in thermal hyperalgesia test (TH) (Figure 1B, unpaired t-test, *n* = 8, t = 6.420, df = 14, *p* < 0.0001) as compared with Wistar rats. WKY rats were genetically resistant to the fluoxetine (10 mg/kg, i. p.) effect on depression (Figure 1C, RM 2-way ANOVA, *n* = 8/group, F (1,14) = 0.399, *p* = 0.538) and hyperalgesia (Figure 1D, RM 2-way ANOVA, *n* = 8/group, F (1,14) = 0.0573, *p* = 0.814) as demonstrated following 14 consecutive days of treatment. (2) In a Wistar rat model of chronic social stress, comorbid depressive behavior (Figure 1E, RM 2-way ANOVA, *n* = 8/group, F (2,21) = 21.8, *p* < 0.0001) shown as increased immobility time in FST (Figure 1E, day 0, Control vs. CSS-Fluo) and hyperalgesia (Figure 1F, RM 2-way ANOVA, *n* = 8/group, F (2,21) = 47.6, *p* < 0.0001) shown as decreased PWL in TH (Figure 1F, day 0, Control vs. CSS-Fluo), were also demonstrated before fluoxetine treatment. Continuous daily treatment with fluoxetine (10 mg/kg i. p.) initially demonstrated antidepressant and antihyperalgesic effects when examined on day 14 shown as the decreased immobility time (Figure 1E, day 14, CSS-Veh vs. CSS-Fluo) and increased PWL (Figure 1F, day 14, CSS-Veh vs. CSS-Fluo). But the fluoxetine effect was substantially reduced when examined on day 28, as the immobility time was again increased (Figure 1E, day 28, Control vs. CSS-Fluo) and the PWL was again decreased (Figure 1F, day 28, Control vs. CSS-Fluo). (3) Wistar rats with CFA-induced inflammatory arthritic pain showed the same reduction of fluoxetine’s antidepressant and antihyperalgesic effects during maintenance treatment (Figures 1G, H, RM 2-way ANOVA, *n* = 8/group; 1G: F (2,21) = 13.7, *p* = 0.0002; 1H: F (2,21) = 81.5, *p* < 0.0001). Collectively, these results from three animal models of comorbid depression and pain demonstrate reduced fluoxetine’s antidepressant and antihyperalgesic effects during maintenance treatment.

### 5-HT1A auto-receptors and Ido1 were upregulated following fluoxetine treatment

**FIGURE 2:**
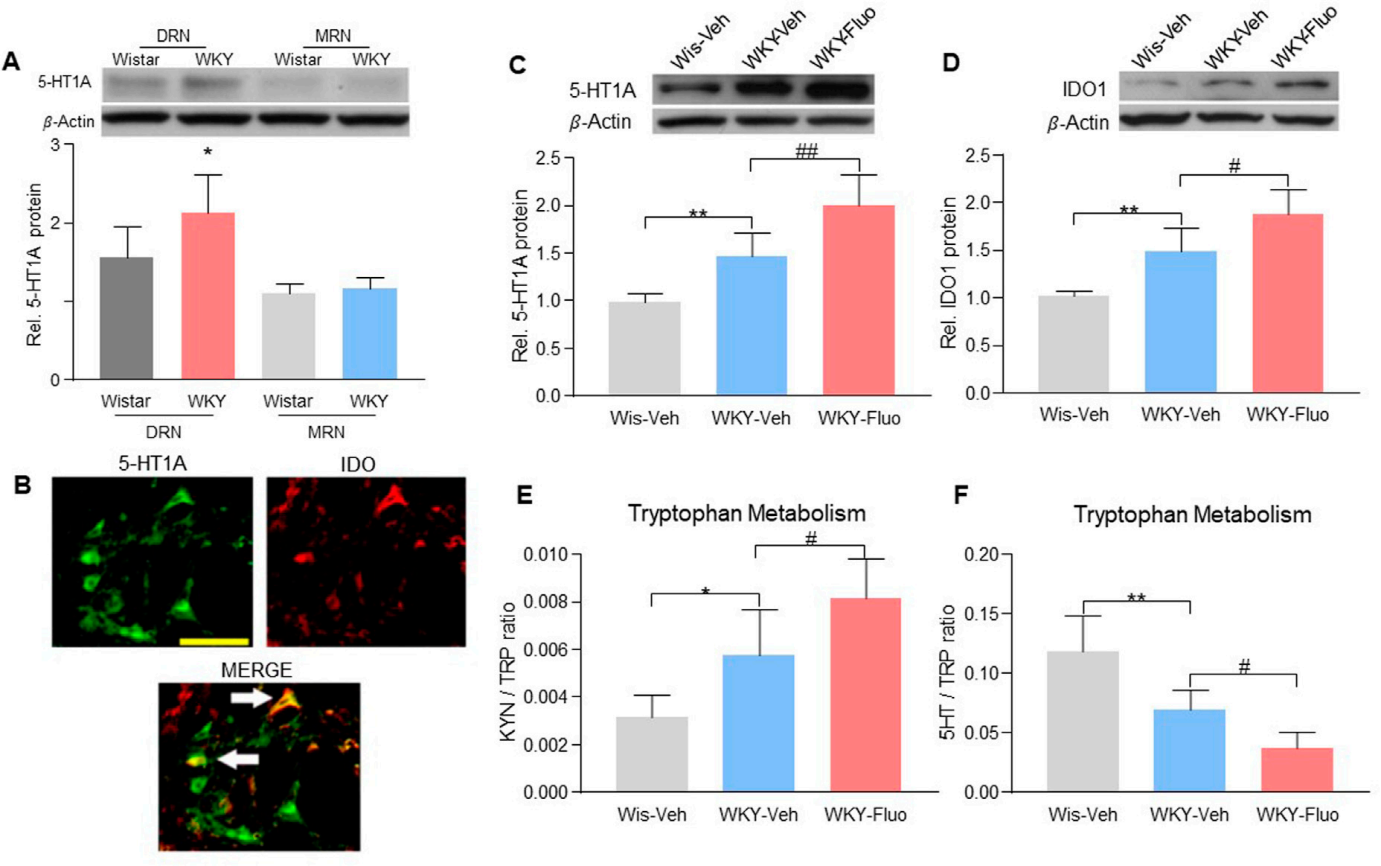
Fluoxetine maintenance treatment increased the expression of 5-HT1A auto-receptors and IDO1 in WKY rats. **(A)** Band of western blot and quantitative analysis showed that the expression of 5-HT1A receptors was increased in the DRN of WKY rats compared to Wistar rats. And there were no differences in the 5-HT1A receptor expression in the MRN between Wistar and WKY rats. β-Actin was included as a control. *p < 0.05 compared to the Wistar-DRN group. **(B)** Immunofluorescence staining showed the colocalization of 5-HT1A receptors and IDO1 in the DRN of WKY rats (scale bar = 100 μm). The arrows indicated the merge of the 5-HT1A receptors and IDO1. Protein expression of 5-HT1A receptors **(C)** and IDO1 **(D)**, as well as IDO1 activity [KYN/TRP ratio **(E)**, HPLC) and 5-HT/TRP ratio **(F)**, HPLC)] were elevated in DRN of WKY rats compared to Wistar rats (WKY-Veh vs. Wis-Veh), and were further increased when treated with fluoxetine (WKY-Veh vs. WKY-Fluo). The data are expressed as the mean ± SD (*n* = 6). *p < 0.05 and **p < 0.01, Wis-Veh vs. WKY-Veh; #*p* < 0.05 and ##*p* < 0.01, WKY-Veh vs. WKY-Fluo. DRN, Dorsal Raphe Nucleus; MRN, Magnus Raphe Nucleus Rel. Indicates relative; Wis, Wistar; Veh, vehicle; Fluo, fluoxetine; KYN, kynurenine; TRP, tryptophan.

We found that 5-HT1A receptors were predominantly expressed in the DRN but not in the magnus raphe nucleus (MRN) in WKY rats (Figure 2A, 2-way ANOVA, *n* = 6/group, F (1,20) = 5.801, *p* = 0.0258). Since IDO1 plays a key role in the comorbidity of pain and depression in our previous study (Kim et al., 2012), we examined whether fluoxetine treatment would change the expression of 5-HT1A auto-receptors and IDO1 in the DRN. Co-localization of 5-HT1A receptors with IDO1 was detected in the DRN of WKY rats (Figure 2B). Further, we found upregulation of 5-HT1A auto-receptors (Figure 2C, one-way ANOVA, *n* = 6/group, F (2,15) = 27.11, *p* < 0.0001) and IDO1 (Figure 2D, one-way ANOVA, *n* = 6/group, F (2,15) = 26.29, *p* < 0.0001) expression in the DRN of WKY rats after 14-days of fluoxetine exposure. Moreover, the ratio of kynurenine (KYN; product of IDO1) over tryptophan (TRP) was increased (Figure 2E, one-way ANOVA, *n* = 6/group, F (2,15) = 15.18, *p* = 0.0002), whereas the ratio of 5-HT over TRP was decreased (Figure 2F, one-way ANOVA, *n* = 6/group, F (2,15) = 21.87, *p* < 0.0001), in the DRN of the rats with upregulated IDO1 expression (Figure 2D), indicating a shift of tryptophan metabolism away from the 5-HT biosynthesis.

### Fluoxetine-induced Ido1 upregulation was dependent on NF-kB regulation

**FIGURE 3:**
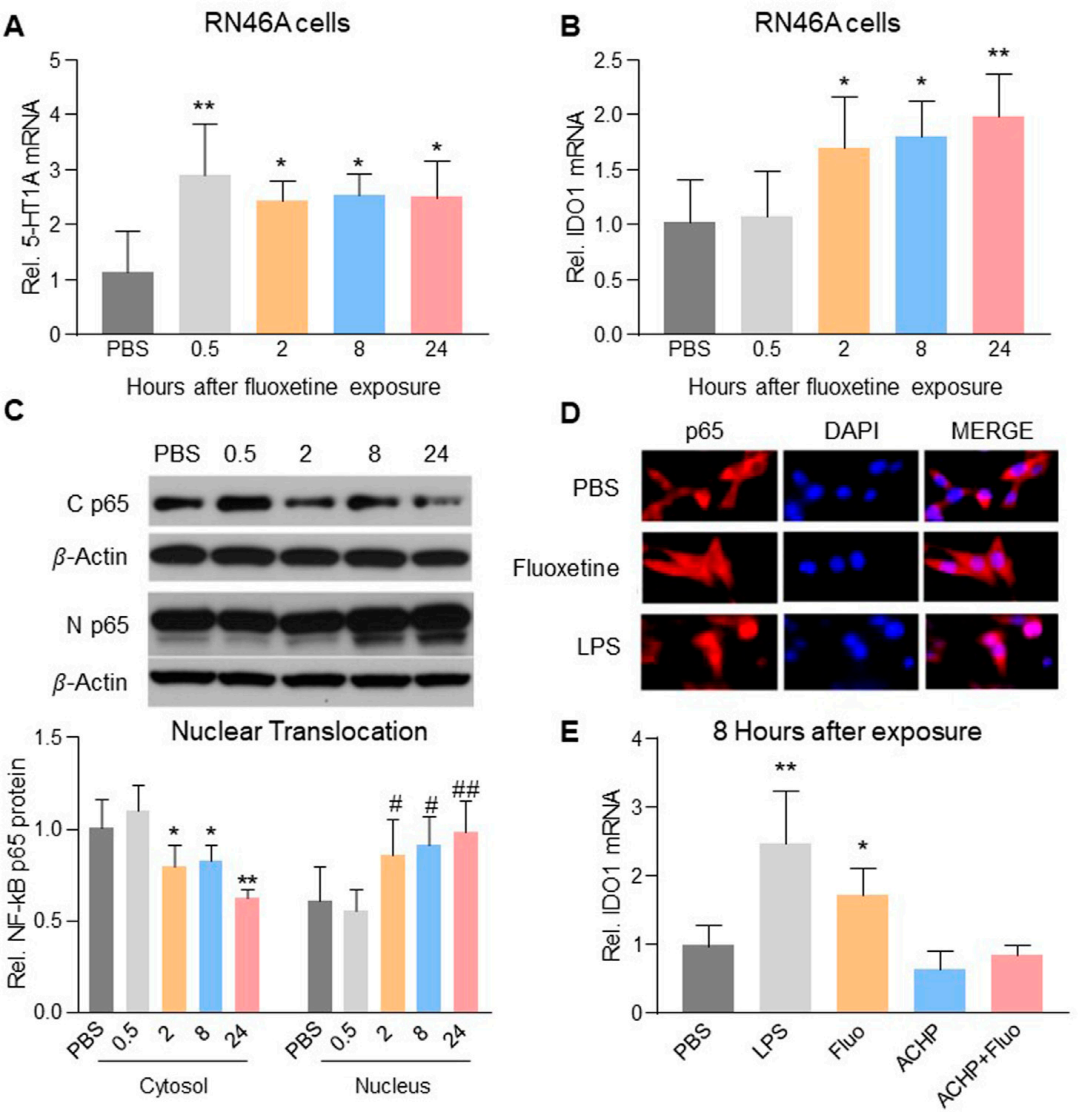
Fluoxetine mediated the upregulation of IDO1 expression *via* NF-*k*B-IDO1 pathway. Fluoxetine upregulated mRNA expression of 5-HT1A receptors **(A)** from 0.5 h and IDO1 **(B)** from 2 h after exposure. **(C)** From 2 h after fluoxetine exposure, the level of NF-*k*B p65 protein in cytosol decreased, but its level in the nucleus increased. **(D)** NF-*k*B p65 immunoreactivity detected in the nucleus (merged with DAPI) was higher in fluoxetine and LPS exposure RN46A cells than in PBS control cells. **(E)** LPS and fluoxetine exposure both significantly increased the mRNA expression of IDO1, which was reversed in ACHP alone and ACHP combined with fluoxetine exposure groups. The data are presented as mean ± SD (*n* = 6). *p < 0.05 and **p < 0.01 compared to the PBS treated group, #*p* < 0.05 and ##*p* < 0.01 compared to the PBS treated group (Nucleus). Rel. Indicates relative; C p65, Cytosol NF-*k*B p65; N p65, Nucleus NF-*k*B p65; Veh, vehicle; Fluo, fluoxetine.

To examine a direct cellular effect of fluoxetine exposure on serotonergic neurons, we exposed cultured RN46A cells, an immortalized serotonergic neuronal cell line, to vehicle (PBS) or fluoxetine (10 μM) (Manjappa et al., 2021) for 0.5, 2, 8, or 24 h. Fluoxetine increased mRNA expression of 5-HT1A receptors (Figure 3A, one-way ANOVA, *n* = 6/group, F (4,25) = 6.116, *p* = 0.0014) and IDO1 (Figure 3B, one-way ANOVA, *n* = 6/group, F (4,25) = 7.364, *p* = 0.0005). Exposure to fluoxetine time-dependently induced the nuclear translocation (Figure 3C, one-way ANOVA, *n* = 6/group, Cytosol: F (4,25) = 15.52, *p* < 0.0001; Nucleus: F (4,25) = 7.713, *p* = 0.0003) of NF-*k*B, a downstream signaling element of 5-HT1A receptors contributory to transcription of IDO (Hemmati et al., 2019) because 1) NF-*k*B p65 immunoreactivity was detected in both the cytosol and nucleus of RN46A cells exposed to fluoxetine or LPS (positive control, 100 *n*g/ml) but only in the cytosol of the PBS control group (Figure 3D), and 2) inhibition of NF-*k*B with ACHP (an IkB kinase inhibitor, 1 μM) (Victoriano et al., 2006) prevented the upregulation of IDO1 in response to fluoxetine exposure (Figure 3E, one-way ANOVA, *n* = 6/group, F (4,25) = 18.42, *p* < 0.0001).

### 5-HT1A auto-receptors were critical to the reduced antidepressant and antihyperalgesic effects of fluoxetine

**FIGURE 4:**
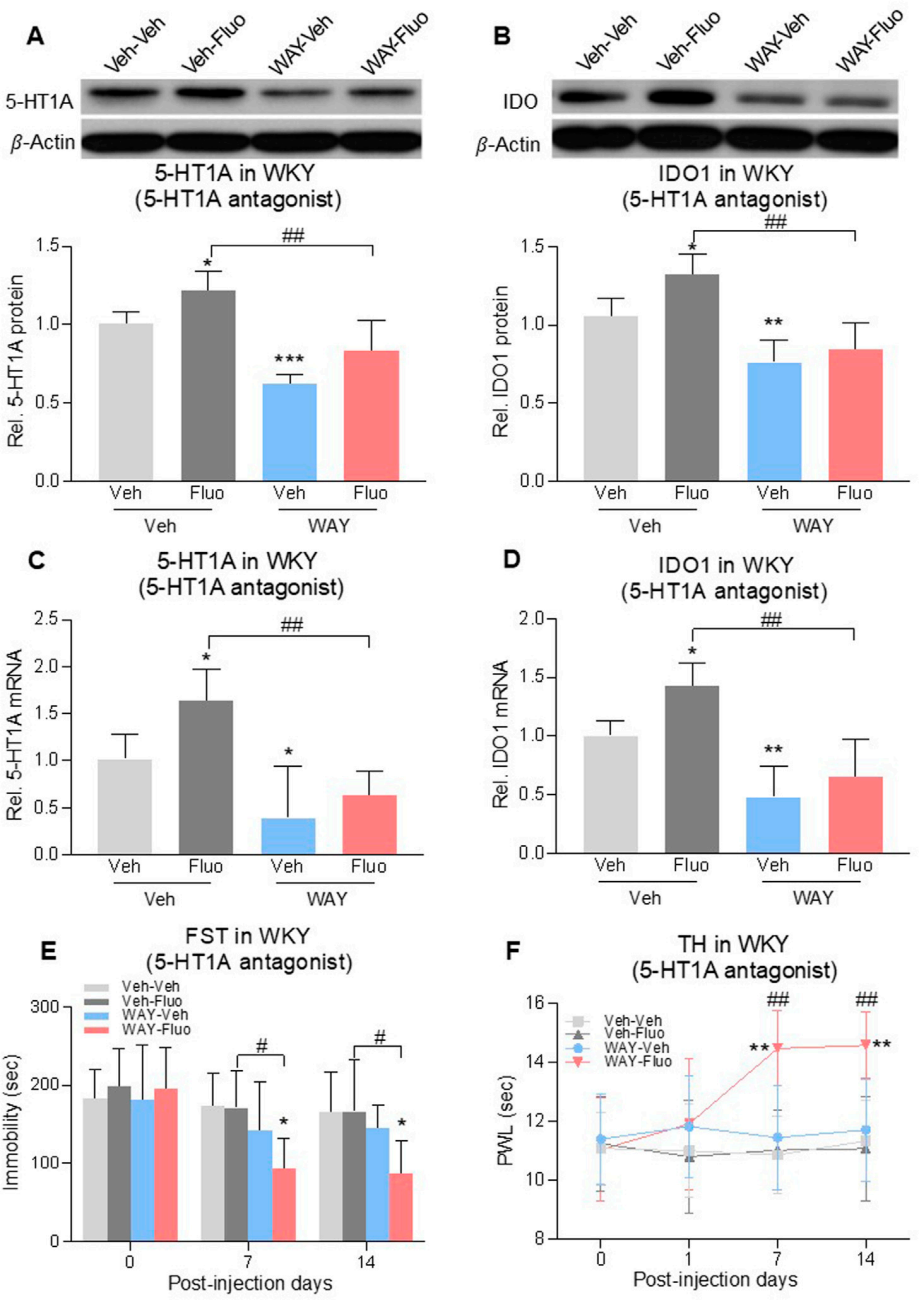
Intra-DRN 5-HT1A receptor antagonist restored the fluoxetine effect in WKY rats. 5-HT1A receptors and IDO1 protein **(A,B)** and mRNA **(C,D)** expression in DRN of WKY rats were increased by fluoxetine (Veh-Veh vs. Veh- Fluo) but reversed by adding intra-DRN WAY (Veh-Fluo vs. WAY-Fluo). Fluoxetine maintenance treatment combined with intra-DRN WAY injection (Veh-Veh vs. WAY- Fluo, Veh-Fluo vs. WAY-Fluo), but not with fluoxetine or WAY alone (Veh-Veh vs. Veh- Fluo or WAY-Veh), decreased immobility time in FST **(E)** and increased PWL in TH **(F)** from day 7–14. The data are presented as mean ± SD (*n* = 6). *p < 0.05, **p < 0.01 and ***p < 0.001 vs. Veh-Veh; #*p* < 0.05 and ##*p* < 0.01, Veh-Fluo vs. WAY-Fluo. Rel. Indicates relative; Veh, vehicle; Fluo, fluoxetine; WAY, WAY100635; FST, forced swim test; TST, tail suspension test; TH, thermal test; PWL, paw withdrawal latency.

To assess the role of 5-HT1A auto-receptors in the reduced antidepressant and antihyperalgesic effects of fluoxetine, we microinjected a selective 5-HT1A receptor antagonist, WAY100635 (1 μg/0.5 μL once daily), into the DRN of WKY rats for 14 days (Carli and Samanin, 2000). Combined treatment of fluoxetine and WAY100635 decreased 5-HT1A auto-receptor and IDO1 expression at both protein levels (Figures 4A, B), 2-way ANOVA, *n* = 6/group, 4A: F (1,20) = 17.05, *p* = 0.0005; 4B: F (1,20) = 9.629, *p* = 0.0056) and mRNA levels (Figures 4C, D, 2-way ANOVA, *n* = 6/group, 4C: F (1,20) = 7.931, *p* = 0.0107; 4D: F (1,20) = 9.902, *p* = 0.0051) that were elevated by fluoxetine exposure. This same treatment regimen also restored the fluoxetine’s antidepressive (Figure 4E, RM 2-way ANOVA, *n* = 6/group, F (3,20) = 3.3, *p* = 0.04) and antihyperalgesic (Figure 4F, RM 2-way ANOVA, *n* = 6/group, F (3,20) = 4.6, *p* = 0.013) effects in WKY rats (Veh-Fluo vs. WAY-Fluo).

**FIGURE 5:**
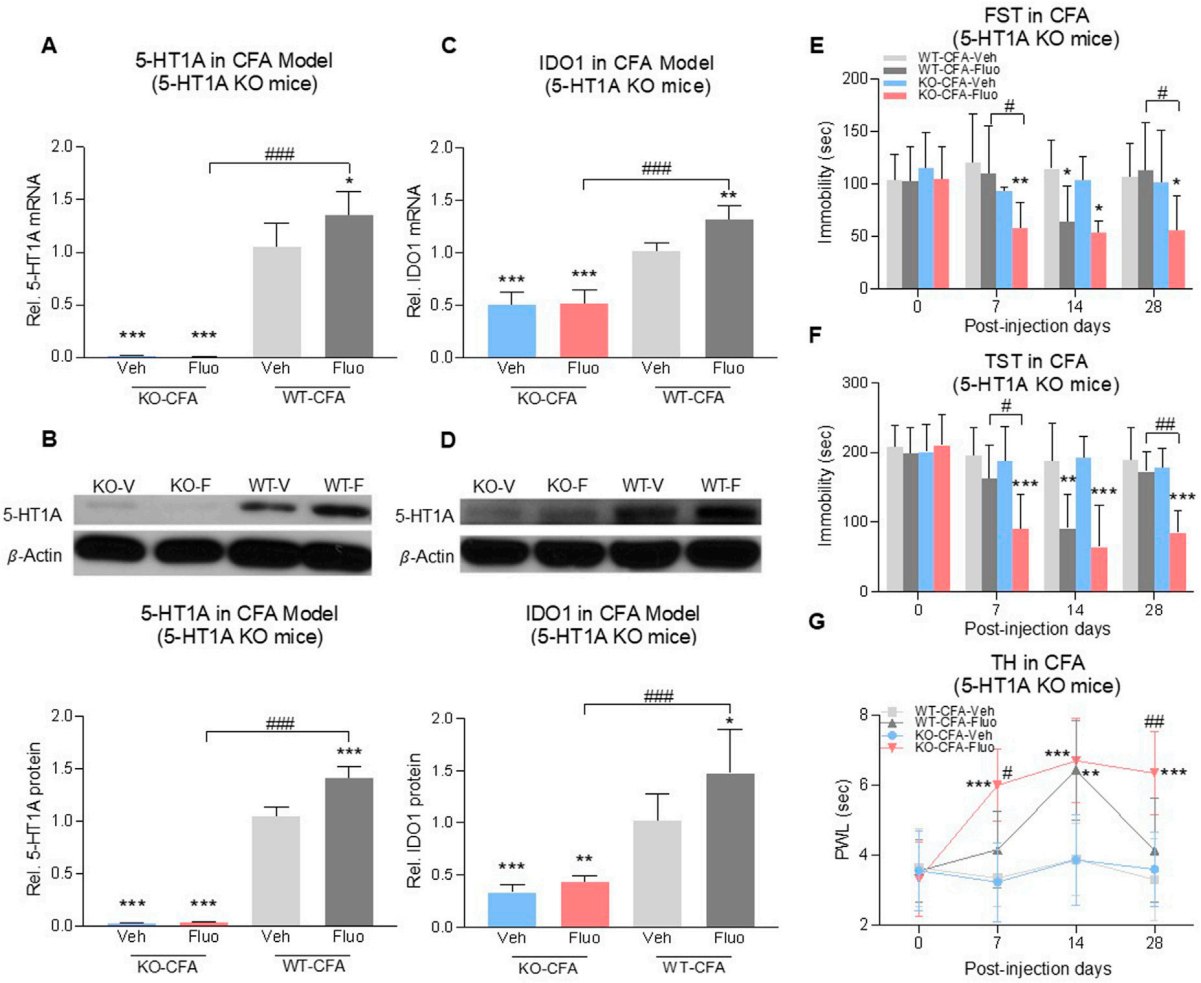
Fluoxetine maintained its antidepressant and analgesic effects in 5-HT1A receptor-KO mice. Fluoxetine increased 5-HT1A receptors **(A,B)** and IDO1 **(C,D)** in WT-CFA model mice (WT-Veh vs. WT-Fluo), while had no effect on KO-CFA model mice (KO-Veh vs. KO-Fluo). **(A,B)** 5-HT1A receptors were absent in 5-HT1A receptor-KO mice (WT-Veh vs. KO-Veh, KO-Fluo). **(C,D)** 5-HT1A receptor-KO decreased IDO1 expression in the DRN (WT-Veh vs. KO-Veh, KO-Fluo). In the CFA model, fluoxetine decreased the immobility time **(E,F)** and increased PWL **(G)** in 5-HT1A receptor-KO mice as early as day 7 (WT-Veh vs. KO-Fluo), as compared to only on day 14 in WT mice (WT-Veh vs. WT-Fluo). 5-HT1A KO maintained chronic fluoxetine antidepressant and antihyperalgestic effects up to day 28 (WT-Fluo vs. KO-Fluo). There was no difference between vehicle-treated KO and vehicle-treated WT mice. The data are presented as mean ± SD (*n* = 6). *p < 0.05, **p < 0.01 and ***p < 0.001 vs. WT-Veh; #*p* < 0.05, ##*p* < 0.01 and ###*p* < 0.001, WT-Fluo vs. KO-Fluo. Rel. Indicates relative; Veh, vehicle; Fluo, fluoxetine; WT, wild type; FST, forced swim test; TST, tail suspension test; TH, thermal test; PWL, paw withdrawal latency.

To further determine the role of 5-HT1A auto-receptors in this process, we used 5-HT1A receptor-knockout (KO) mice (Figure 5B, 2-way ANOVA, *n* = 6/group, 5A: F (1,20) = 5.298, *p* = 0.0322; 5B: F (1,20) = 43.13, *p* < 0.0001) with CFA-induced comorbid pain and depression. 5-HT1A receptor-KO showed the reduced expression of IDO1 in the DRN at the baseline (Figures 5C, D), 2-way ANOVA, *n* = 6/group, 5C: F (1,20) = 10.01, *p* = 0.0049; 5D: F (1,20) = 7.325, *p* = 0.0136) (WT-Veh vs. KO-Veh, WT-Fluo vs. KO-Fluo). Fluoxetine treatment increased the expression of 5-HT1A receptors and IDO1 in the DRN of wild-type mice (WT-Veh vs. WT-Fluo), but not in 5-HT1A KO mice (Figures 5A–D) (KO-Veh vs. KO-Fluo). Consistently, 5-HT1A KO mice demonstrated the sustained antidepressant and antihyperalgesic effects following 28 consecutive days of fluoxetine treatment (Figures 5E–G) and also an earlier onset (on day 7) of the antidepressant (Figures 5E, F), RM 2-way ANOVA, *n* = 6/group, 5E: F (3,20) = 4.57, *p* = 0.01; 5F: F (3,20) = 11.08, *p* = 0.0002) and antihyperalgestic (Figure 5G, RM 2-way ANOVA, *n* = 6/group, F (3,20) = 10.1, *p* = 0.0003) effects as compared with WT mice under the same fluoxetine treatment regimen. There were no differences in depressive and hyperalgesic behaviors between WT-Veh group mice and KO-Veh group mice (Figures 5E–G).

### Inhibition of Ido1 also restored the antidepressant and antihyperalgesic effects of fluoxetine

**FIGURE 6:**
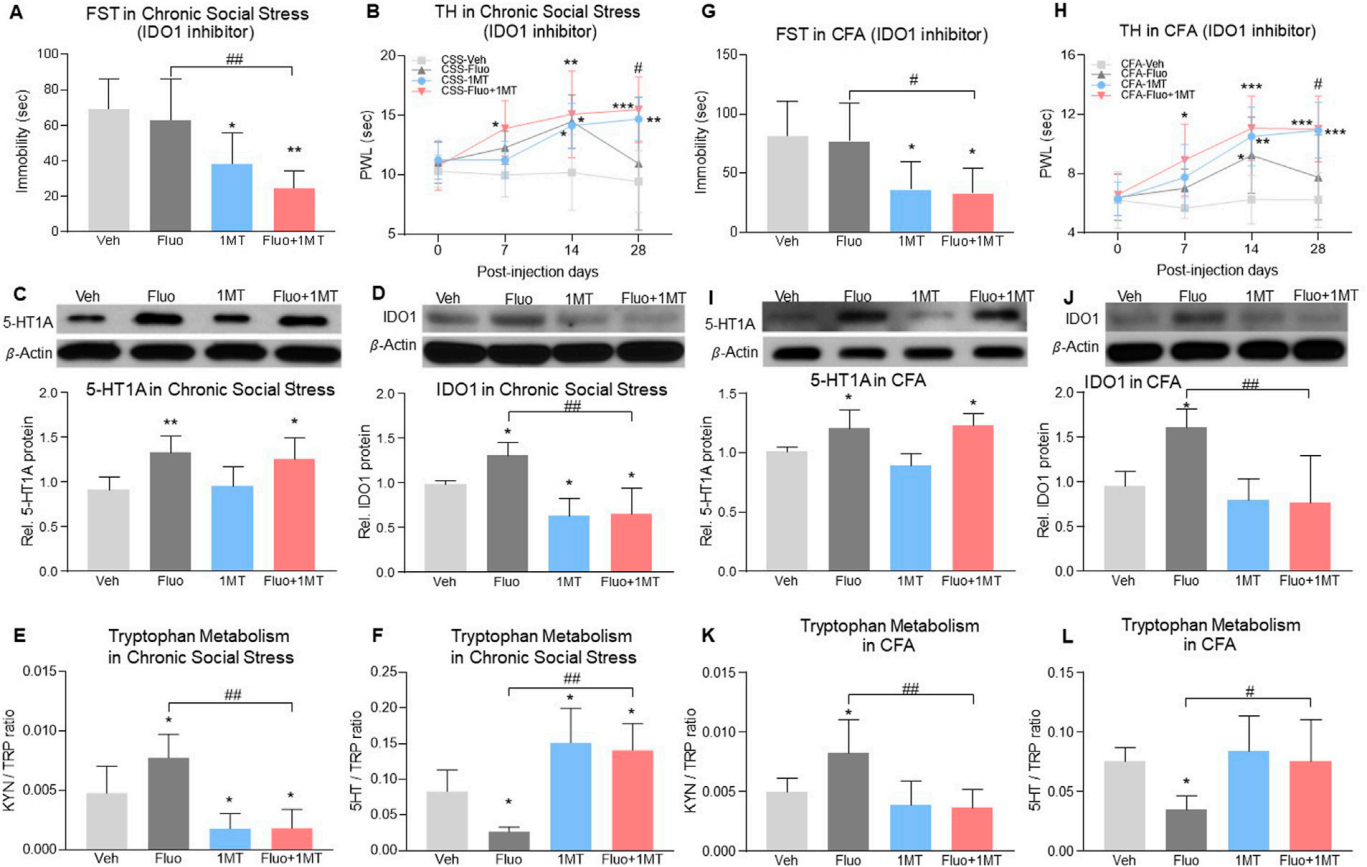
Inhibition of IDO1 activity maintained fluoxetine’s antidepressant and antihyperalgesic effect. **(A)** In the chronic social stress model, treatment with 1MT alone or in combination with fluoxetine decreased the immobility time in FST on day 28 as compared to vehicle-treated rats. **(B)** PWL was increased by fluoxetine or 1MT alone from day 14 but fell back to baseline on day 28 in fluoxetine alone group (CSS-Veh vs. CSS-Fluo or CSS-1MT). Combined with 1MT, fluoxetine increased PWL from day 7 (CSS-Veh vs. CSS-Fluo+1MT)and maintained its antihyperalgestic effects up to day 28 (CSS-Fluo vs. CSS-Fluo+1MT) **(C,D)** Expression of 5-HT1A receptors and IDO1 in the DRN was increased by fluoxetine maintenance treatment in chronic social stress rats as compared to vehicle-treated rats (CSS-Veh vs. CSS-Fluo). 1MT alone (CSS-Veh vs. CSS-1MT) or in combination with fluoxetine (CSS-Veh vs. CSS-Fluo+1MT, CSS-Fluo vs. CSS-Fluo+1MT) decreased the protein level of IDO1 **(D)** but 5-HT1A receptors **(C)**. **(E,F)** 1MT alone or in combination with fluoxetine reduced the IDO activity in the DRN, which was elevated by fluoxetine alone. **(G,H)** In the CFA model, 1MT maintained antidepressant and antihyperalgestic effects of fluoxetine up to day 28 (CFA-Fluo vs. CFA-Fluo+1MT). 1MT combined with fluoxetine or alone treatment increased PWL from day 7 and day 14 respectively as compared to vehicle-treated CFA rats. Fluoxetine alone increased PWL on day 14 only (CFA-Veh vs. CFA-Fluo). **(I–L)** 1MT decreased expression and activity of IDO1 (CFA-Fluo vs. CFA-Fluo+1MT), which prevented fluoxetine from increasing expression and activity of IDO1 (CFA-Veh vs. CFA-Fluo). The data are presented as mean ± SD (*n* = 6). *p < 0.05, **p < 0.01 and ***p < 0.001 compared to Veh-treated group; #*p* < 0.05 and ##*p* < 0.01, CSS-Fluo vs. CSS-Fluo+1MT, CFA-Fluo vs. CFA-Fluo+1MT. FST, forced swim test; TH, thermal test; PWL, paw withdrawal latency; Rel. Indicates relative; Veh, vehicle; Fluo, fluoxetine; KYN, kynurenine; TRP, tryptophan.

To examine whether 5-HT1A-mediated IDO1 upregulation would be important for the reduced fluoxetine therapeutic effects, we administered L-1-methyl-tryptophan (1-MT, IDO1 inhibitor, 10 mg, i. p.) once daily for 28 consecutive days according to a previous report (O'Connor et al., 2009) in models of comorbid pain and depression. In chronic social stress rats with comorbid pain and depression, 1MT alone or in combination with fluoxetine decreased immobility time in FST when examined on day 28 as compared to vehicle treated group (Figure 6A, 2-way ANOVA, *n* = 6/group, F (1,20) = 23.41, *p* < 0.0001). The effect of combination treatment was more effective than the fluoxetine or 1MT alone treatment, shown as combined treatment with fluoxetine and 1-MT increased PWL in TH as early as day 7 (Figure 6B, RM 2-way ANOVA, *n* = 6/group, F (3,20) = 5.76, *p* = 0.005) and maintained the fluoxetine’s antidepressant and antihyperalgesic effects up to day 28 (Figures 6A, B). Moreover, 1-MT did not change the upregulation of 5-HT1A auto-receptors (Figure 6C, 2-way ANOVA, *n* = 6/group, F (1,20) = 0.02633, *p* = 0.8727) but reduced the expression of IDO1 (Figure 6D, 2-way ANOVA, *n* = 6/group, F (1,20) = 41.36, *p* < 0.0001), supporting the notion that IDO1 upregulation was downstream to the 5-HT1A upregulation induced by fluoxetine exposure. Importantly, inhibition of IDO1 with 1-MT also restored normal kynurenine/tryptophan and 5-HT/tryptophan ratios that were altered by fluoxetine exposure (Figures 6E, F), 2-way ANOVA, *n* = 6/group, *p* < 0.0001, 6E: F (1,20) = 35.87, 6F: F (1,20) = 42.75). Similar outcomes were observed in rats with CFA-induced comorbid pain and depression in which 1-MT combined with fluoxetine prevented the reduced antidepressant and antihyperalgesic effects and restored normal kynurenine/tryptophan and 5-HT/tryptophan ratios induced by fluoxetine exposure alone (Figures 6G–L, 2-way ANOVA, *n* = 6/group, 6G: F (1,20) = 16.14, *p* = 0.0007, 6I: F (1,20) = 1.246, *p* = 0.2775, 6J: F (1,20) = 14.69, *p* = 0.0010, 6K: F (1,20) = 12.67, *p* = 0.0020, 6L: F (1,20) = 6.013, *p* = 0.0235; 6H: RM 2-way ANOVA, *n* = 6/group, F (3,20) = 6.27, *p* = 0.0036).

## Discussion

**FIGURE 7:**
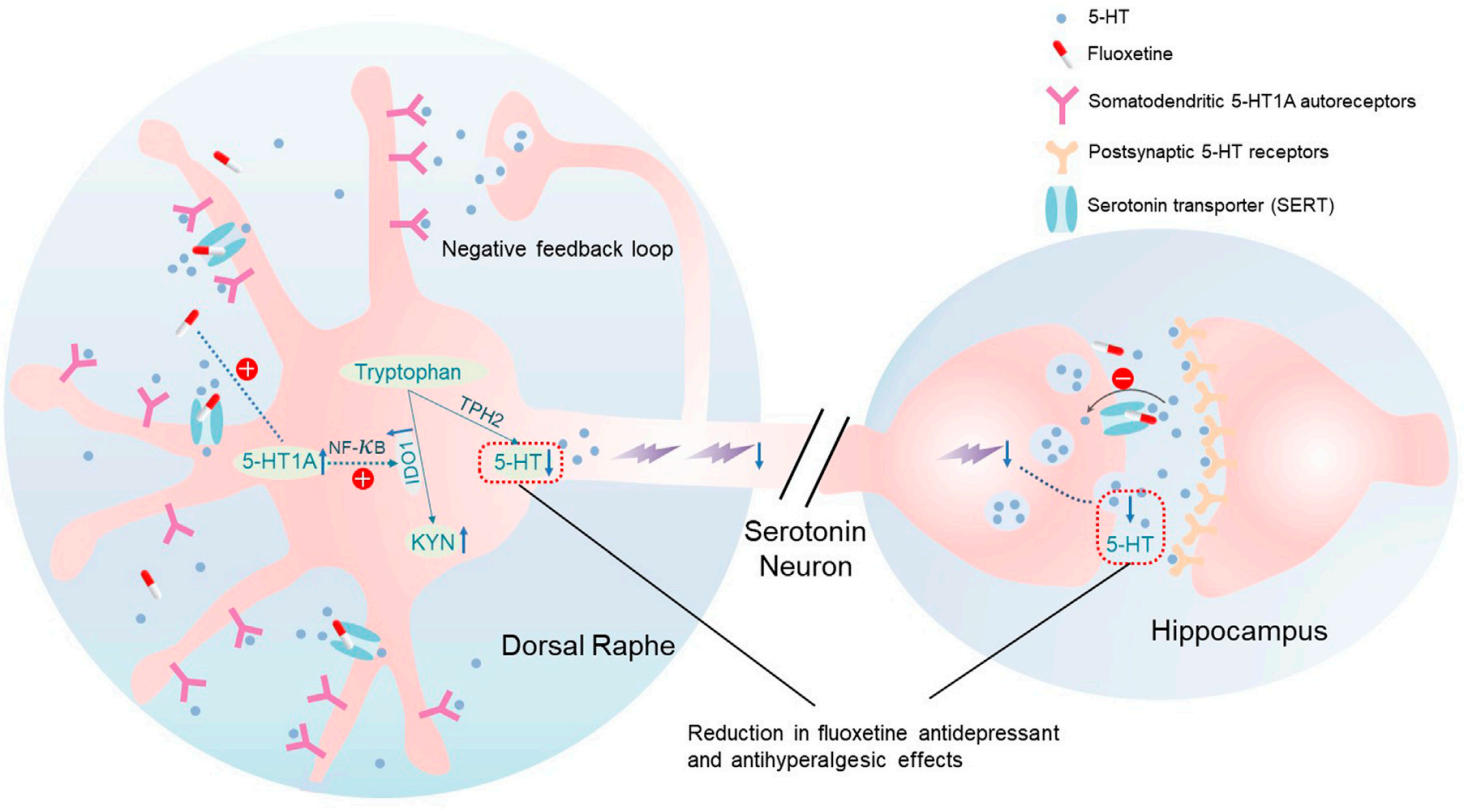
Schematic of the reduction in fluoxetine effects.

In three comorbid pain and depression animal models (genetic predisposition, chronic social stress, arthritis), we demonstrated 1) the reduced antidepressant and antihyperalgesic effects of fluoxetine during its maintenance treatment, 2) upregulation of 5-HT1A and IDO1 expression in the DRN following fluoxetine exposure, 3) inhibition or knockout of 5-HT1A receptors prevented the fluoxetine-induced upregulation of IDO1 expression, and 4) inhibition of either the 5-HT1A receptor or IDO1 activity maintained the fluoxetine’s antidepressant and antihyperalgesic effects. Collectively, our results indicate that 5-HT1A-mediated upregulation of IDO1 in the DRN is a critical mechanism for the reduced antidepressant and antihyperalgesic effects of fluoxetine (Figure 7).

WKY rats are genetically predisposed to depression and hyperalgesia (Sundaram et al., 2022) and resistant to fluoxetine treatment (Kim et al., 2012). Consistently, we observed resistance to fluoxetine treatment in WKY rats. Reduced fluoxetine’s antidepressant and antihyperalgesic effects was also demonstrated in rats with chronic social stress-or CFA-induced comorbid pain and depression, indicating a broad implication of this phenomenon across several comorbid pain and depression conditions. As shown in this study, WKY rats showed depression-like and hyperalgesic behaviors when compared to Wistar rats (Figures 1A, B). Consistently, WKY rats had the higher 5-HT1A protein level in the DRN than Wistar rats (Figures 2A, C). WKY rats did not respond to fluoxetine (Figures 1C, D), whereas the 5-HT1A antagonist improved fluoxetine’s effects as early as Day 7 in WKY rats (Figures 4E, F). Taken together, resistance to fluoxetine in WKY rats is likely due to an elevated 5-HT1A baseline level. However, fluoxetine exposure may have further increased 5-HT1A expression in WKY rats (Figure 2C). As such, the 5-HT1A antagonist improved the fluoxetine effects in WKY rats (Figures 4E, F), which is consistent with the role of 5-HT1A auto-receptors in the reduced effects of fluoxetine as observed in both chronic social stress and CFA models.

The 5-HT1A receptor is a subtype of serotonergic receptors. Activation of this receptor has been implicated in the mechanisms of anxiolytic, antidepressant and antipsychotic medications (Bantick et al., 2004; Newman-Tancredi, 2010). As a primary source of 5-HT in the brain, the DRN is critical for the mechanisms of action of SSRIs. Antidepressants regulate 5-HT neuronal firings in the raphe nuclei (Crespi, 2010). Accumulating evidence indicates that 5-HT1A receptors participate in the antinociceptive effects of fluoxetine (Berrocoso and Mico, 2009; Cervantes-Duran et al., 2013; Obata, 2017) and regulate serotonergic neuronal firings in the DRN (Llado-Pelfort et al., 2012). Although the exact mechanism underlying the effect of SSRIs on the serotonergic circuit in the DRN remains unclear, SSRIs such as fluoxetine have been shown to decrease 5-HT-mediated neuronal activities in the DRN and 5-HT release in the brain regions of serotonergic projections such as the hippocampus (Rainer et al., 2012; Moncrieff et al., 2022).

5-HT1A receptors located on somatodendritic sites of serotonergic neurons in the DRN are known to be auto-receptors and activation of 5-HT1A auto-receptors inhibit the firing of serotonergic neurons and release of 5-HT at synaptic sites (Slifirski et al., 2021). It has been reported that 5-HT1A receptor desensitization is required for the onset of SSRI efficacies, which is consistent with the fact that deletion of 5-HT1AR induces a decrease in immobility time in FST (Ramboz et al., 1998). However, the increased 5-HT1A receptor was observed with chronic fluoxetine treatment (28 days) in our study. This discrepancy is likely due to the different time point at which the 5-HT1A was measured. It has been reported that on Day 14 of chronic fluoxetine treatment in rats, density of 5-HT1A receptors and their coupling with G proteins in the DRN were decreased, and neuronal firing in the DRN was inhibited (Le Poul et al., 2000; Hanoun et al., 2004). Accordingly, the 5-HT1A receptor might have decreased before Day 14 while fluoxetine remained effective in our chronic social stress and CFA models, and the 5-HT1A receptor was subsequently increased on Day 28 when the fluoxetine effects was gone. This observation further verified the hypothesis stated in other’s study that the desensitization of presynaptic 5-HT1A receptor function on dorsal raphe neurons may underlie the therapeutic efficacy of long-term SSRI treatment (Pejchal et al., 2002; Babb et al., 2018). In this study, we focused on Day 28 fluoxetine treatment to further explore the mechanism of subsequent loss of fluoxetine efficacy. We showed that upregulation of 5-HT1A auto-receptors in the DRN induced by fluoxetine exposure decreased the 5-HT/tryptophan ratio in the DRN. We further demonstrated that IDO1 expression was also upregulated in the DRN, which was downstream to the 5-HT1A upregulation an NF-kB-dependent intracellular regulatory pathway that involves translation of NF-kB from the cytosol to the nucleus (Abdouh et al., 2004). The prolonged use of SSRIs induces high levels of extracellular 5-HT, which can lead to the activation of 5-HT1A auto-receptors (Figure 7). This negative feedback loop is believed to be the cause of the delayed clinical efficacy of antidepressant drugs (Artigas et al., 2001; Guilloux et al., 2006). Our results further indicate that blocking or knocking-down 5-HT1A auto-receptors accelerated the onset of the antidepressant and antihyperalgestic effects of fluoxetine and maintained the fluoxetine’s antidepressant and antihyperalgestic effects during maintenance treatment. Similarly, inhibition of IDO1 with 1-MT also maintained the fluoxetine’s antidepressant and antihyperalgestic effects during maintenance treatment. These observations suggest a negative 5-HT1A-IDO1 regulatory pathway in the DRN (Audero et al., 2008), which can be activated by fluoxetine exposure and is contributory to the reduced antidepressant and antihyperalgesic effects of fluoxetine by shifting the tryptophan metabolism toward the KYN/TRY pathway with progressive reduction of 5-HT synthesis (Okaty et al., 2019).

Kynurenine and serotonin are two major tryptophan metabolites. IDO1, a rate-limiting enzyme in tryptophan metabolism, is located in the kynurenine production pathway and alteration of IDO1 expression changes the content of endogenous kynurenine and 5-HT, such that an increase in IDO1 activity lowers the endogenous 5-HT level (Hoglund et al., 2019), which leads to depression (Zhou et al., 2019) and diminished descending inhibition of pain modulation (Rojewska et al., 2018). Our previous study has shown that regulation of brain IDO1 by proinflammatory cytokines serves as a critical mechanism of comorbid pain and depression through regulating tryptophan metabolism (Kim et al., 2012). SSRIs have been shown to exert the antidepressant effect by shifting the balance of the KYN/TRY pathway toward the 5-HT/TRY pathway in hippocampus (Wang et al., 2022). In this study, for the first time, we found that fluoxetine increased the IDO1 expression and decreased the 5-HT/TRY ratio in the DRN concurrently with the loss of its antidepressant and antihyperalgesic effects. Moreover, our data showed that the fluoxetine effects were maintained by inhibiting IDO1 activity with 1-MT, which also normalized KYN/TRY and 5-HT/TRY ratios. It should be pointed out that 1-MT alone improved the antidepressant and antihyperalgestic effects in chronic social stress and CFA models, which could be due to the upregulated IDO1 expression in chronic social stress and CFA models in the absence of fluoxetine exposure as reported in others (Gabbay et al., 2012; Salazar et al., 2012) and our own study (Kim et al., 2012). However, combined treatment with fluoxetine and 1-MT accelerated the antihyperalgestic effects and maintained the fluoxetine’s antidepressant and antihyperalgesic effects during maintenance treatment, suggesting that the combined fluoxetine and 1-MT regimen could be a new strategy to enhancing and maintaining the effects of SSRI.

It has been extensively studied that the antidepressant-like response to fluoxetine (Gomez et al., 2014) and other SSRIs, like paroxetine (David et al., 2001) or sertraline (Kokras et al., 2015), varies according to sex. Alonso, et al. reported that, in middle-aged rats, female rats showed a clear antidepressant-like response to fluoxetine at 5–10 mg/kg, while male rats only responded to fluoxetine at 10 mg/kg (Fernandez-Guasti et al., 2017). Clinical observations also showed a better response of women versus men in the antidepressant-like effect of fluoxetine (Martenyi et al., 2001). Such sexual differentiation in SSRIs response may be due to a dissimilarity of 5-HT1A receptor activity. In PET studies, women have significantly higher 5-HT1A receptor and lower 5-HT transporter (5-HTT) binding potentials in the brain than men (Jovanovic et al., 2008). In the hypothalamus and DRN of 5-HTT mutant mice, the reduction of 5-HT1A receptors is more extensive in female than in male mice, which demonstrates a strong correlation between 5-HT1A receptor and 5-HTT binding potentials in female mice (Li et al., 2000). Female rodents also exhibited a greater sensitivity to 5-HT1A agonists compared to male rodents (Blanchard et al., 1992). Although the reduced efficacy of SSRI occurs in both sexes (Katz, 2011; Amsterdam and Lorenzo-Luaces, 2018), potential sex differences across these effects with regards to dose regimens and molecular mechanisms are yet to be determined. Since only male rodents were used in this study, female rodents need to be included in further research to examine any sex effect in the reduced antidepressant and antihyperalgesic effects of fluoxetine during maintenance treatment.

In summary, our results reveal a novel role of 5-HT1A auto-receptor-mediated IDO1 upregulation in the DRN in the reduced antidepressant and antihyperalgesic effects of fluoxetine. These preclinical findings could warrant clinical investigations into the possibility of blocking 5-HT1A auto-receptors and/or IDO1 as a strategy to maintain the therapeutic effects of SSRI for the treatment of comorbid pain and depression in clinical settings.

## Data Availability

The raw data supporting the conclusions of this article will be made available by the authors, without undue reservation.
